# High levels of oncomiR-21 contribute to the senescence-induced growth arrest in normal human cells and its knock-down increases the replicative lifespan

**DOI:** 10.1111/acel.12069

**Published:** 2013-04-19

**Authors:** Hanna Dellago, Barbara Preschitz-Kammerhofer, Lucia Terlecki-Zaniewicz, Carina Schreiner, Klaus Fortschegger, Martina W-F Chang, Matthias Hackl, Rossella Monteforte, Harald Kühnel, Markus Schosserer, Florian Gruber, Erwin Tschachler, Marcel Scheideler, Regina Grillari-Voglauer, Johannes Grillari, Matthias Wieser

**Affiliations:** 1 Department of Biotechnology, BOKU-VIBT University of Natural Resources and Life Sciences ViennaMuthgasse 18, 1190, Vienna, Austria; 2 CCRI - Children's Cancer Research InstituteZimmermannplatz 10, 1090, Vienna, Austria; 3 Institute of Physiology, Pathophysiology and Biophysics, Department of Biomedical Sciences, University of Veterinary Medicine ViennaVeterinärplatz 1, A-1210, Vienna, Austria; 4 Department of Dermatology, Medical University of ViennaA-1090, Vienna, Austria; 5 C.E.R.I.E.S. - Centre de Recherches et d'Investigations Epidermiques et Sensorielles20 Rue Victor Noir, 92200, Neuilly-sur-Seine, France; 6 Institute for Genomics and Bioinformatics, Graz University of TechnologyPetersgasse 14, 8010, Graz, Austria; 7 Evercyte GmbHMuthgasse 18, 1190, Vienna, Austria; 8 Austrian Centre of Industrial Biotechnology (ACIB GmbH)Muthgasse 18, 1190, Vienna, Austria

**Keywords:** CDC25A, CDK2, cellular senescence, hyperoncogenic signal, microRNA, miR-21, NFIB, p21

## Abstract

Cellular senescence of normal human cells has by now far exceeded its initial role as a model system for aging research. Many reports show the accumulation of senescent cells *in vivo*, their effect on their microenvironment and its double-edged role as tumour suppressor and promoter. Importantly, removal of senescent cells delays the onset of age-associated diseases in mouse model systems. To characterize the role of miRNAs in cellular senescence of endothelial cells, we performed miRNA arrays from HUVECs of five different donors. Twelve miRNAs, comprising hsa-miR-23a, hsa-miR-23b, hsa-miR-24, hsa-miR-27a, hsa-miR-29a, hsa-miR-31, hsa-miR-100, hsa-miR-193a, hsa-miR-221, hsa-miR-222 and hsa-let-7i are consistently up-regulated in replicatively senescent cells. Surprisingly, also miR-21 was found up-regulated by replicative and stress-induced senescence, despite being described as oncogenic. Transfection of early passage endothelial cells with miR-21 resulted in lower angiogenesis, and less cell proliferation mirrored by up-regulation of p21^CIP1^ and down-regulation of CDK2. These two cell-cycle regulators are indirectly regulated by miR-21 via its validated direct targets NFIB (Nuclear factor 1 B-type), a transcriptional inhibitor of p21^CIP^^1^, and CDC25A, which regulates CDK2 activity by dephosphorylation. Knock-down of either NFIB or CDC25A shows a phenocopy of over-expressing miR-21 in regard to cell-cycle arrest. Finally, miR-21 over-epxression reduces the replicative lifespan, while stable knock-down by sponges extends the replicative lifespan of endothelial cells. Therefore, we propose that miR-21 is the first miRNA that upon its knock-down extends the replicative lifespan of normal human cells.

## Introduction

Cellular senescence is an irreversible growth arrest of normal somatic cells that is caused by telomere shortening, oxidative stress, DNA damage or by hyperoncogenic signalling (Ben-Porath & Weinberg, 2005). Thus, cellular senescence has been proposed to be a potent tumour suppressive mechanism that prohibits potentially damaged and genetically instable cells from further division via either p16^INK4A^ or p21^CIP1^ as key executers of cell-cycle arrest (Ben-Porath & Weinberg, 2005). Indeed, senescence has been found to act as a tumour suppressor in mouse models *in vivo* (Cosme-Blanco *et al*., 2007; Feldser & Greider, 2007). Similarly, hyperoncogenic signalling by H-RAS has been found to induce senescence in normal cells unless a second cooperating oncogene is present (Serrano *et al*., 1997).

However, antagonistic pleiotropic effects of senescence have been reported as well. As senescent cells seem to persist and accumulate with age in different tissues (Campisi & Sedivy, 2009), their altered functional profile including a pro-inflammatory secretory phenotype changes the tissue microenvironment in ways that can promote both cancer and aging phenotypes (Krtolica & Campisi, 2002). This view is supported by the findings that removal of senescent cells (Baker *et al*., 2011) or re-elongation of telomeres (Jaskelioff *et al*., 2011) in transgenic mice can delay the onset of age-related pathologies. These functions of senescence have recently been summarized as the ‘four faces’ of senescence (Rodier & Campisi, 2011).

To better understand senescence and how the senescent phenotype of cells might contribute to aging or age-associated pathologies, we have been investigating the cellular senescence-related changes in gene and protein expression profiles of various cell types (Chang *et al*., 2005; Laschober *et al*., 2010). We found that similar to fibroblasts also endothelial cells acquire a senescence-associated secretory phenotype (eSASP), secreting high levels of pro-inflammatory cytokines like, for example interleukin-8 (Hampel *et al*., 2006). With regard to how the senescent phenotype is induced and maintained, only a few reports, however, have focused on the role of miRNAs (reviewed in Grillari & Grillari-Voglauer, 2010; Smith-Vikos & Slack, 2012; Schraml & Grillari, 2012) but still little is known on how miRNAs interact with cell-cycle regulators and functionally contribute to the senescent phenotype.

Here, we report the expression profiling of miRNAs in endothelial cell senescence using human umbilical vein endothelial cells (HUVEC) from 5 individual donors. We identified 12 miRNAs to be up-regulated in senescence, comprising hsa-miR-23a, hsa-miR-23b, hsa-miR-24, hsa-miR-27a, hsa-miR-29a, hsa-miR-31, hsa-miR-100, hsa-miR-193a, hsa-miR-221, hsa-miR-222 and hsa-let-7i. Surprisingly, also miR-21 was consistently up-regulated in replicative senescence as well as in oxidative stress–induced premature senescence (SIPS). Mimics of miR-21 introduced into early passage cells induce a cell-cycle arrest by targeting CDC25A and NFIB that in turn down-regulate CDK2 and induce p21^CIP1^. Since miR-21 is one of the first oncomiRs that have been reported (Si *et al*., 2007), these data suggest that in normal somatic cells miR-21 alone is sufficient to provide a hyperoncogenic signal limiting aberrant proliferation.

## Results

### miR-21 is up-regulated in senescent HUVECs

**Fig. 1:**
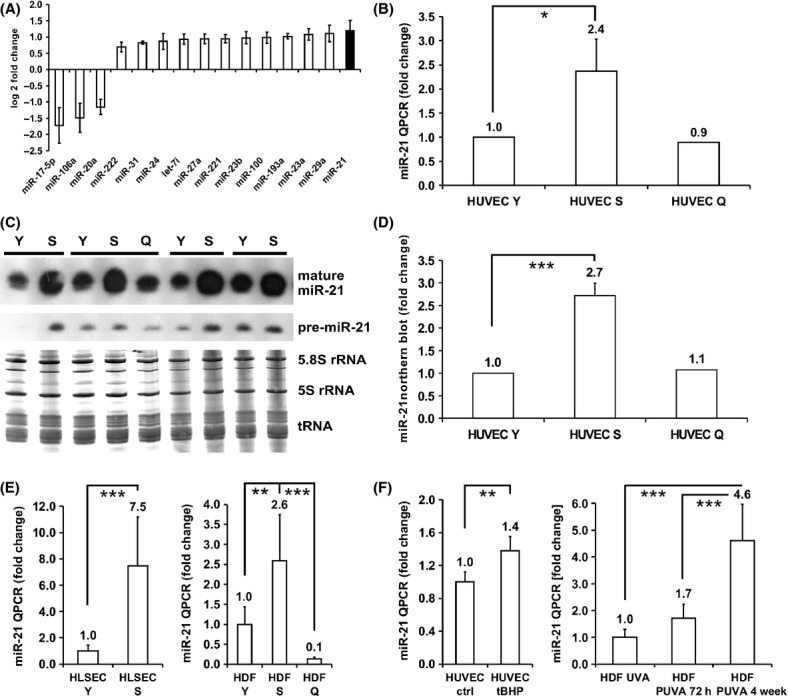
miR-21 is up-regulated in cellular senescence. (A) Micro-RNA microarray expression profiling of five senescent HUVEC strains, showing miR-21 as the miRNA with the strongest induction in senescent HUVECs. Log2-fold changes relative to young counterparts are indicated. (B) qPCR taqman analysis of miR-21 of the 5 HUVEC strains. (C) Northern blot analysis of HUVEC strains (4 out of 5; for one donor RNA amounts were limiting) confirms miR-21 induction in senescent cells on the mature and pre-miRNA level. (D) Quantification of Northern blot analysis of the different senescent HUVEC strains normalized to 5S rRNA, and relative to early passage cells. (E) miR-21 is up-regulated in senescent human liver sinusoidal endothelial cells (HLSEC; left panel) and in human dermal fibroblasts (HDF; right panel) as analysed by qPCR. (F) miR-21 is up-regulated in stress-induced senescence. Senescence was induced in HUVEC by repeated tert-butyl hydroperoxide (tBHP) treatment (left panel), and in HDF by psoralen and UVA treatment (PUVA; right panel). All experiments were independently repeated at least 3 times. Y, early passage; S, senescent; Q, quiescent (**P < 0.01 *t*-test). Error bars represent mean ± standard deviation. Statistical analysis was performed using student's *t*-test. In cases where three groups were compared, statistical analysis was performed using anova with Bonferroni as *post hoc* test. *P*-values indicated represent *P < 0.05; **P < 0.01; ***P < 0.001.

To compare miRNA expression of early passage vs. replicatively senescent HUVECs Exiqon LNA microarray technology was used. We found 12 miRNAs (hsa-miR-21, hsa-miR-23a, hsa-miR-23b, hsa-miR-24, hsa-miR-27a, hsa-miR-29a, hsa-miR-31, hsa-miR-100, hsa-miR-193a, hsa-miR-221, hsa-miR-222 and hsa-let-7i) that were consistently up-regulated in the senescent cells of all donors (Fig. 1A), whereas only three miRNAs of the 17–92 cluster were down-regulated (Fig. 1A). Data have been submitted and are available at array express (E-MEXP-3596), and the senescent status of all 5 HUVEC strains was extensively characterized (Fig. S1).

Microarray expression analysis was confirmed using independent methods (Fig. 1B–D and S2). These findings are consistent with results of HUVEC strains from four different donors published recently (Hackl *et al*., 2010). Since more miRNAs were up-regulated than down-regulated, we also tested if this shift towards up-regulation of miRNA expression in senescence might be due to increased expression of the miRNA-processing protein Dicer in senescent cells. This does not seem to be likely, since Dicer mRNA was down-regulated in senescent cells by 2-fold (Fig. S3A).

From the 12 up-regulated miRNAs, we decided to follow-up on miR-21 being the most prominent miRNA up-regulated in senescence. First, we confirmed that expression levels were significantly (*P* < 0.05) higher in senescent HUVECs by qPCR in all 5 donors (Fig. 1B) and by Northern blot analysis (Fig. 1C,D). The latter shows that the pre-miR-21 is also up-regulated, suggesting transcriptional control of miR-21 in senescence. To see whether such an up-regulation of miR-21 in senescence is a general phenomenon, we tested other replicatively senescent cell types like human liver sinusoidal endothelial cells (HLSEC) and human dermal fibroblasts (HDF; Fig. 1E). Since miR-21 levels in quiescent cells resemble the levels of exponentially growing cells (Fig. 1B–E), the observed up-regulation seems senescence specific in all cell types tested. In addition, stress-induced senescence of either HUVECs after repeated tBHP (*tert*-Butyl hydroperoxide) treatment or fibroblasts after PUVA (psoralen plus UVA) treatment resulted in up-regulation of miR-21 (Fig. 1F). Four weeks after PUVA treatment, when the senescent growth arrest was permanently established, miR-21 expression was significantly elevated. This trend was already observed 72 h after treatment, although it was not statistically significant at that time point.

These data suggest that miR-21 might be generally up-regulated in cellular senescence and might play a role in either its induction or maintenance. The observed increase in miR-21 expression was in all cases statistically significant.

### miR-21 over-expression induces growth arrest

**Fig. 2:**
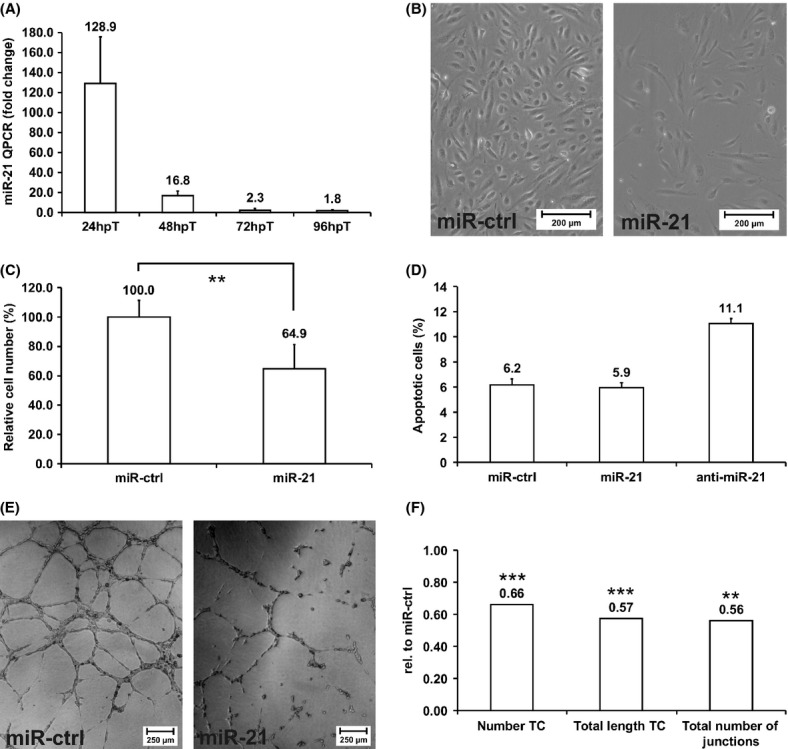
Transient over-expression of miR-21 in HUVECs induces a senescent-like phenotype. (A) qPCR taqman analysis of miR-21 fold changes 24–96 h post-transfection (hpT) of young HUVECs with a miR-21 mimetic. MiR-21 expression was normalized to snRNA-U6 and fold change was calculated relative to cells transfected with a nontargeting control miRNA. (B) Representative image of HUVEC cell density and morphology 48 h post-transfection with a control (left panel) or miR-21 mimetic (right panel). (C) Relative MTT signal of miR-21 transfected HUVEC in per cent of MTT signal measured in control transfected cells 48 hpt (*n* = 6). (D) MiR-21 over-expressing and control transfected HUVEC exhibit a similar extent of apoptosis 48 hpt, whereas transfection with an antimiR targeting miR-21 increases the percentage of apoptotic cells from 6% to 12%. Analysis was performed by flow cytometry (*n* = 3). Cells staining positive for Annexin-V and negative for propidium iodide were considered apoptotic. (E) Representative image of the effect of miR-21 over-expression on the neo-angiogenic potential of young HUVECs analysed by matrigel assay. (F) Quantification of number and total length of tubule complexes (TC) as well as number of junctions. Data from at least 6 randomly chosen images of 3 independent miR-21 and control transfections was analysed using the Angioquant software. Values relative to cells transfected with a nontargeting control are displayed.

To test whether miR-21 over-expression induces a senescence-like growth arrest, a synthetic miR-21 mimic was transfected into early passage HUVECs. Elevated levels of miR-21 for at least 72 h after transfection were confirmed by TaqMan-assays (Fig. 2A). The consequences of miR-21 over-expression in early passage HUVECs were a reduced proliferation and an overt change in morphology as compared to cells transfected with a scrambled control miRNA. The phenotypic changes were most obvious 48 h after transfection (Fig. 2B), and at that time, the effect of miR-21 on cell proliferation was quantified. Using MTT assay as a measure of cell numbers, we observed a highly significant reduction by more than 35% as compared to control (Fig. 2C). To verify that the decrease in cell numbers is due to reduced proliferation and not to increased cell death, we determined apoptosis in miR-21 over-expressing cells. While no differences in the levels of apoptotic cells were observed after miR-21 transfection, the use of an antagomiR to inhibit endogenous miR-21 increased in the percentage of apoptotic cells, from 6% to 12% (Fig. 2D). Furthermore, miR-21 transfected HUVECs displayed a decreased neo-angiogenic potential similar to what we observed in senescent cells expressing high levels of endogenous miR-21 (Fig 2E,F and S1E). Therefore, miR-21 over-expression mimics various aspects of cellular senescence of endothelial cells.

### miR-21 over-expression induces cellular senescence

To further investigate the effect of miR-21 on the cellular lifespan, we transfected HUVECs with lentiviral particles encoding hsa-miR-21 or a nontargeting control shRNA and selected stable clones. Successful transduction was easily monitored by flow cytometry, since GFP was expressed under the control of the same promoter as miR-21.

**Fig. 3:**
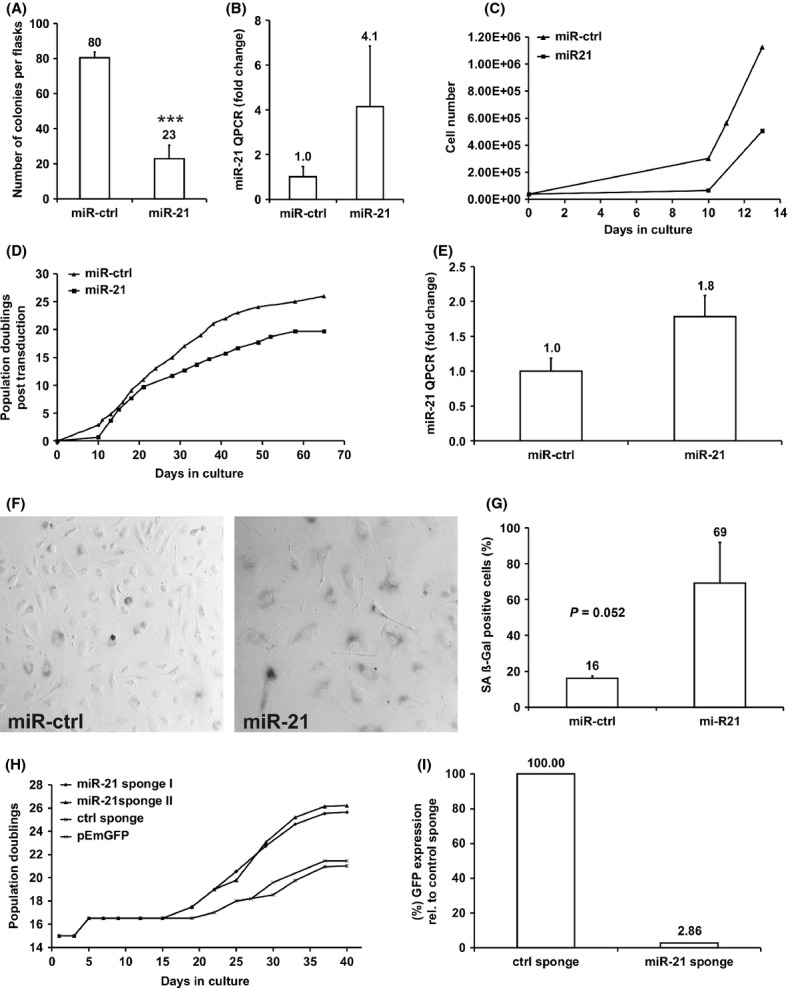
Stable miR-21 over-expression regulates growth potential and senescence. (A) HUVEC were transduced with lentiviral particles over-expressing miR-21 or a nontargeting control miR. Selection pressure was applied and colonies were counted at day 10 post-transduction. Error bars represent mean ± standard deviation obtained from three biological replicates. Statistical analysis was performed using Student's *t*-test. *P*-values indicated represent *P < 0.05; **P < 0.01; ***P < 0.001. (B) qPCR TaqMan analysis of miR-21 fold changes of stable miR-21 over-expressing HUVEC at PD1pT. miR-21 expression was normalized to snRNA-U6, and fold change was calculated relative to cells transfected with a nontargeting control shRNA. (C) HUVEC cell number was determined before seeding for transduction and at two or three passages post-transduction for miR-21 and miR control, respectively. (D) Growth curves of stable miR-21 and miR control over-expressing HUVEC. Population doublings at the first three passages were calculated according to cell numbers and, at later passages, according to split ratios. (E) qPCR taqman analysis of miR-21 fold changes of stable miR-21 over-expressing HUVEC at PD7pT. MiR-21 expression was normalized to snRNA-U6 and fold change was calculated relative to cells transfected with a nontargeting control miRNA. (F) SA-ß-gal staining was carried out at the first passage post-transduction. Representative pictures of miR-21 and miR control over-expressing HUVEC are shown. (G) Quantitation of SA-ß-gal staining. Error bars represent mean ± standard deviation of three biological replicates. For each replicate, at least 10 visual fields at 100-fold magnification were counted. (H) Growth of stable miR-21 sponge expressing HUVEC. HUVEC were transfected with either a miR-21 complete complementary sponge, a scrambled sequence sponge, or the corresponding empty vector. After selection of stable clones, population doublings were calculated according to cell numbers counted at each passage. (I) Percentage of cells expressing GFP fused to the miR-21 sponge was normalized to GFP expression in control sponge HUVEC.

We generated 2 independent stable miR-21 over-expressing and 2 miR-scrambled sequence control HUVEC lines (referred to as miR-21 and miR control HUVEC, respectively). Using the same MoI of virus, miR-21 over-expression significantly reduced colony-forming ability of HUVECs in terms of numbers of colonies (Fig. 3A) as well as size of colonies (data not shown) as compared to HUVECs stably transduced with miR control. To follow-up on this initial indication that stable miR-21 over-expression reduces the proliferative capacity, we determined cell numbers when selection was completed (referred to as PD1pT) and at two subsequent passages. Stable miR-21 over-expression (Fig. 3B) indeed significantly reduced cell proliferation (Fig. 3C) consistent with the transient over-expression results above (Fig. 2C). Cell growth was further monitored during the entire replicative lifespan, and indeed, miR-21 over-expressing cells underwent replicative senescence earlier than control cells (Fig. 3D). Furthermore, miR-21 over-expression led to an increase in senescent cells, as the majority of cells stain positive for senescence-associated ß-galactosidase (SA ß-gal) at PD1pT after selection of stable cells (Fig. 3F,G). However, some cells resume growth, undergo additional 19 population doublings and finally reach replicative senescence at PD20pT, while control cells keep proliferating up to PD26pT. Sustained miR-21 over-expression, albeit lower than after selection of the cells, at later PDLs was confirmed by qPCR (Fig. 3E). This suggests that the amount of miR-21 impacts on the proliferative capacity of the cells.

Then, we tested if knock-down of miR-21 by sequestration using a completely complementary sponge would lead to extension of the replicative lifespan. This is indeed the case (Fig. 3H). The miR-21 sponge was transcribed as a fusion with the 3′-UTR of a GFP cassette. Accordingly, binding of miR-21 results in cleavage of the GFP mRNA allowing to monitor functionality of the miR-21 sponge by GFP suppression (Fig. 3I).

Taken together, these results indicate that miR-21 levels modulate the replicative lifespan of normal human cells.

### Stable miR-21 over-expression induces p21^CIP1^ and pCDK2 as effectors of growth arrest

When we analysed predicted targets of miR-21, we found CDC25A and NFIB, both of which have recently been confirmed as targets of miR-21 (Fujita *et al*., 2008; de Oliveira *et al*., 2009; Wang *et al*., 2009). CDC25A is a phosphatase removing an inhibitory phosphate residue at Thr14 and Tyr15 from CDK2 (Fernandez-Vidal *et al*., 2008), whereas NFIB is a transcriptional repressor of p21^CIP1^ (Ouellet *et al*., 2006).

**Fig. 4:**
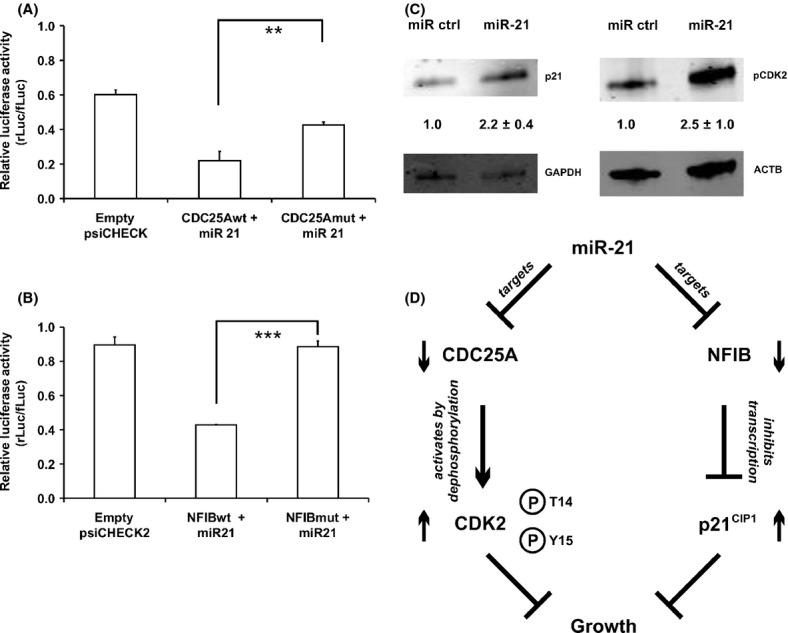
miR-21 regulates cell-cycle progression via its targets CDC25A and NF1B. (A) CDC25A and NF1B are direct targets of miR-21. The expression of a luciferase reporter gene containing the CDC25A 3′-UTR (A) or NF1B 3′-UTR (B), respectively, was suppressed by miR-21 in HUVEC. The suppression is specific to the miR-21 seed region within the CDC25A 3′-UTR or NF1B 3′-UTR, as mutation of the miR-21 target sites alleviated repression by miR-21. Ratios of CDC25A- or NF1B-dependent renilla luciferase signal (rLuc) to a firefly luciferase signal (fLuc) encoded by the same vector are given. *P < 0.05; **P < 0.01; ***P < 0.001. (C) Stable miR-21 or miR control over-expressing HUVEC at PD1 post-transduction were harvested and lysed in SDS loading dye. Samples were subjected to Western blotting with antibodies against pCDK2 and p21^CIP1^, respectively. p21^CIP1^ and pCDK2 were increased in stable miR-21 HUVEC. Quantitation of bands was carried out using the Odyssey software (Licor) Values indicated are mean ± standard deviation obtained from three biological replicates. (D) Model of miR-21 dependent proliferation arrest. miR-21 targets 2 complementary pathways that converge on cell-cycle arrest. MiR-21 (left side) knocks-down its direct target CDC25A. Resulting low levels of the phosphatase CDC25A are insufficient for removing inhibitory phosphate residues from CDK2, thus inhibiting cell-cycle progression. In the second branch (right side), miR-21 directly knocks-down NFIB, which is a transcriptional repressor of p21^CIP1^. Low levels of NFIB in turn allows p21^CIP1^ protein levels to increase and inhibit cell proliferation.

Using a dual reporter assay that allows normalization for intraexperimental variations, we confirmed that also in HUVECs CDC25A (Fig. 4A) and NFIB (Fig. 4B) are direct targets of miR-21.

In consequence, the downstream effectors of NFIB and CDC25A, p21^CIP1^ and pCDK2, respectively, are up-regulated upon mir-21 over-expression (Fig. 4C). Thus, miR-21 over-expression in early passage HUVECs showed the expected effect on miR-21 targets NFIB and CDC25A, along with a corresponding regulation of their respective downstream targets, 21^CIP1^ and phospho-CDK2, reminiscent of that of senescent cells.

These results suggest that the miR-21 induced growth arrest might be independent of well-acknowledged regulators of senescence-like p53 and p16. Indeed, no differential transcription of these mRNAs was observed (Fig. S4A). To confirm independence of p53, we used the same lentiviral particles for miR-21 and control miR with HeLa cells, in which p53 is highly instable due to rapid E6-oncoprotein-dependent degradation (May *et al*., 1991). Strikingly, miR-21 over-expressing Hela cells formed significantly less colonies during selection of stable clones, and colonies were also significantly smaller (Fig.S4B,C), even though transduction efficiency, measured as percentage of GFP-positive cells 48-h post-transduction, was higher for miR-21 than for miR-control (Fig. S4D). However, we cannot exclude that miR-21 acts downstream of p53. While p53 is supposed to induce miR-21 in a p53-inducible cancer cell line (Tarasov *et al*., 2007), no correlation between miR-21 and p53 expression was observed in cancer tissues (Rask *et al*., 2011; Mathé *et al*., 2012). However, taken together our data suggest a model for miR-21-induced growth arrest via the two branches of CDK2 and p21^CIP1^ (Fig. 4D).

### CDC25A and NFIB knock-down phenocopies the effect of miR-21 over-expression in HUVECs

**Fig. 5:**
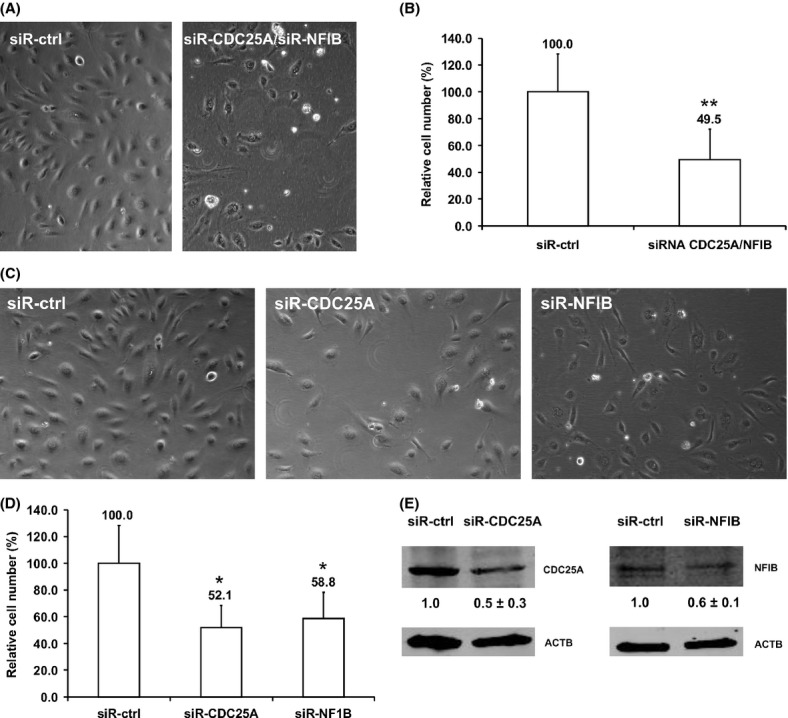
Knock-down of the p21^CIP^^1^ and CDK2 regulators NFIB and CDC25A inhibit proliferation. (A) Similar to miR-21 over-expression, simultaneous knock-down of both, the CDK2 regulator CDC25A and the p21^CIP1^ regulator NFIB using siRNA smartpools induce a growth arrest as shown by a representative image 48 h after transfection. (B) Quantification of the observed growth arrest by visual evaluation of the average cell number in 6 randomly chosen microscopic fields. (C) Individual knock-down of either CDC25A or NFIB reduce proliferation as shown in a representative image 48 h after siRNA transfection. (D) Quantification of the observed growth arrest by visual evaluation of the average cell number in 6 randomly chosen microscopic fields. (E): Confirmation of protein reduction after knockdown. CDC25A siRNAs reduce protein levels as by Western blotting (left panel). siRNA-mediated NFIB knock-down reduces NFIB protein levels (right panel).

To confirm that the growth arrest of miR-21 over-expressing cells is indeed executed via CDC25A and NF1B, we knocked down CDC25A and NF1B to phenocopy miR-21 over-expression. To this end, we transiently transfected early passage HUVECs with siRNAs against NFIB and CDC25A. Indeed, knockdown of both CDC25A and NFIB inhibited proliferation of HUVECs as compared to cells transfected with a control siRNA. Forty-eight hours after transfection a reduction in cell number by 50% was observed by quantification of randomly chosen microscopic fields (Fig. 5A,B).

Furthermore, we tested the effect of individually knocking down either CDC25A or NFIB. Surprisingly, either of these knock-downs was sufficient to induce cell-cycle arrest (Fig. 5C,D). Efficient knock-down of CDC25A and NFIB was evident on protein level by reduced CDC25A and NFIB protein (Fig. 5E).

These data support the hypothesis that miR-21 targets two branches of cell-cycle inhibitory pathways that individually are sufficient to reduce cell proliferation. Interfering with both branches enhances the growth inhibitory effect. We suggest that miR-21 by targeting separate branches converging on the same final outcome of cellular behaviour (as in this case growth arrest) increases the robustness of cellular response to stimuli.

### miR-21 is up-regulated upon stress-induced senescence *in vivo*

**Fig. 6:**
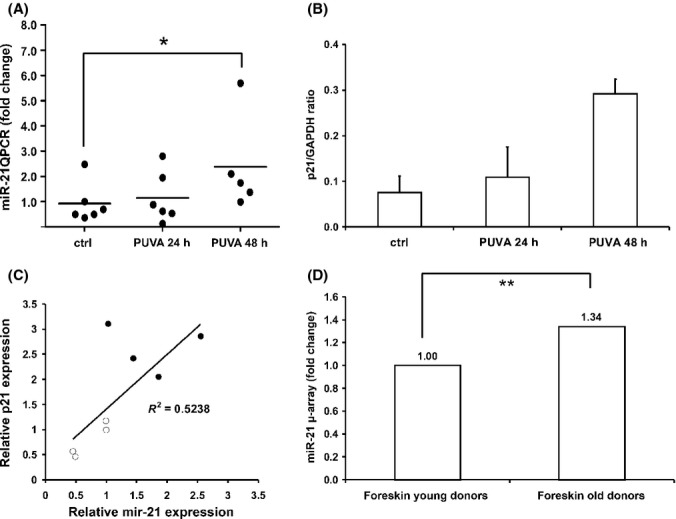
PUVA treatment induces miR-21 in mouse skin *in vivo*. (A) Analysis of *in vivo* induction of miR-21 in the back skin of mice 24 and 48 h after PUVA treatment using qPCR using at least 5 C57Bl/6 mice. Expression was normalized to snoRNA-202 as house-keeping control. A significant increase in miR-21 was observed 48 h after treatment (*P* < 0.05; Mann-Whitney test). (B) qPCR analysis of p21^CIP1^ mRNA levels after PUVA treatment as normalized to GAPDH mRNA. (C) Correlation of p21^CIP1^ to miR-21 expression. (D) miR-21 is also up-regulated in skin of elderly donors using μ-array analysis. Data were re-analysed and visualized from Hackl *et al*., 2010; in which also correlative up-regulation of p21^CIP1^ in the foreskin of elderly has been shown.

PUVA is combined treatment of the photosensitizer psoralen and UV-A radiation. This treatment is used against psoriasis and various other dermatoses and results in premature skin aging *in vivo* and in cellular senescence *in vitro* (reviewed in Wlaschek *et al*., 2003). Therefore, we exposed C57BL/6mice to PUVA doses reported to induce cellular senescence *in vivo* (Singh *et al*., 2010). Forty-eight hours after treatment, we observed a significant induction of miR-21 using at least 5 animals per group (Fig. 6A). The miR-21 up-regulation was concomitant with p21^CIP1^ up-regulation (Fig. 6B), and the relative expression of p21^CIP1^ to miR-21 correlated quite well (*r*^2^ = 0.52). These data are well in line with our previous observation that miR-21 is significantly up-regulated in foreskin of old vs. young donors (Fig. 6D, data re-plotted from supplements of (Hackl *et al*., 2010) as well as p21^CIP1^ levels as published recently (Hackl *et al*., 2010).

Taken together, our data suggest that miR-21 is induced in normal human cells undergoing replicative or stress-induced senescence *in vitro* and *in vivo* and contributes to inducing and/or maintaining the senescence-associated growth arrest.

## Discussion

Here, we report that miR-21, one of the most prominent onco-miRs, is up-regulated during replicative and stress-induced senescence of human umbilical cord vein endothelial cells, sinusoidal liver endothelial cells as well as in human dermal fibroblasts. Since senescent endothelial cells are present *in vivo* at sites of atherosclerosis (reviewed by (Erusalimsky, 2009), this seems well in agreement with high levels of miR-21 in human atherosclerotic plaques (Raitoharju *et al*., 2011).

However, one recent report found down-regulation of miR-21 in senescent human aortic endothelial cells (Weber *et al*., 2010), and it remains to be elucidated if such differences are due to differences of the endothelial cell type or cultivation conditions.

In any case, several findings connect miR-21 to the cardiovascular system. Mir-21 is up-regulated upon various types of stress in rat vascular smooth muscle cells (VSMCs) (Ji *et al*., 2007; Lin *et al*., 2009), in cardiomyocytes after H_2_O_2_ exposure (Cheng *et al*., 2009), as well as *in vivo*, in rat carotid arteries after balloon injury (Ji *et al*., 2007), but also in human VSMCs exposed to hypoxia (Sarkar *et al*., 2010) or to ischaemic preconditioning (Dong *et al*., 2009) and in endothelial cells after shear stress (Weber *et al*., 2010). In all these settings, miR-21 up-regulation inhibits apoptosis and induces cell proliferation of VSMCs, contributing to the formation of neointima thickening *in vivo*.

Thus, in VSMCs and cardiomyocytes induction of miR-21 expression upon stress leads to cellular phenotypes resembling an anti-apoptotic and pro-proliferative effect that fits miR-21's role as an oncomiR. Indeed, neointimal lesion formation in proliferative vascular disease shares many similarities with cancers (Ross *et al*., 2001).

In contrast, when testing the effect of miR-21 over-expression on endothelial cell proliferation, we found an antiproliferative effect of miR-21, which is supported by similar findings by Fleissner (Fleissner *et al*., 2010). Although this observation may at first seem counterintuitive, one has to be aware of the well-known fact that miRNAs can have different effects in different cell types [reviewed by (Calin & Croce, 2006; Esquela-Kerscher & Slack, 2006)].

Since miR-21 is high in senescence, our results suggest that it might contribute to inducing or maintaining the senescent cell-cycle arrest (Fig. 5F). Strikingly, our findings that two separate pathways converging on endothelial cell-cycle arrest by targeting p21^CIP1^ and CDK2 as key effectors support such an idea. Indeed, p21^CIP1^ is known to be transcriptionally repressed by NFIB (Ouellet *et al*., 2006), which is an experimentally validated target of miR-21 (Fujita *et al*., 2008), so that high miR-21 decreases NFIB and p21^CIP1^ is de-repressed. The second regulatory branch is by miR-21 directly targeting the phosphatase CDC25A (de Oliveira *et al*., 2009; Wang *et al*., 2009). Thereby, the cell-cycle inhibitory phosphorylation on CDK2 cannot be removed and reduces the cell-cycle promoting activity of CDK2 (Shen & Huang, 2012). This scheme is well in line with the recently proposed model of endothelial cell senescence, where a prominent role of reduction of CDK2 protein levels has been postulated (Freedman & Folkman, 2005).

These findings still seem to be very counterintuitive to the a plethora of reports showing that miR-21 over-expression is involved in carcinogenesis and tumour progression (Jazbutyte & Thum, 2010).

In our opinion, these paradoxical data might be reconciled by the hypothesis that miR-21 acts and/or contributes to induce senescence triggered by hyperoncogenic signalling in normal cells similar to oncogenic Ras (Serrano *et al*., 1997).

This idea is supported by the fact that H-RAS induces miR-21 (Loayza-Puch *et al*., 2010). It is intriguing to think that in this type of tumour, onco-miR-21-induced cellular senescence might slow down the disease progression, as by now hyperoncogenic-signalling-induced senescence has been shown to be a tumour suppressor mechanism *in vivo* in mouse models. Another similarity is shared by the type of tumour that is formed in H-RAS/MYC transgenic mice or in miR-21 transgenic mice. Both specifically develop B-cell lymphoma (Langdon *et al*., 1989; Medina *et al*., 2010).

Taken together, we suggest that miR-21 is a cellular senescence- induced miRNA that contributes to induction and/or maintenance of a senescence-like cell-cycle arrest in normal human cells. Since overwhelming evidence identifies miR-21 as a bona fide onco-miR, this cell-cycle arrest might be due to hyperoncogenic signalling similar to the effect of over-expressing oncogenic H-RAS in normal human cells.

## Experimental procedures

### Cell culture

Human umbillical cord vein endothelial cells (HUVEC) were isolated as described previously (Jaffe *et al*., 1973) and cultivated using EGM medium (Lonza, Basel, Switzerland) supplemented with foetal calf serum (FCS) to 10%. Human liver sinusoidal endothelial cells were grown in EGM-2 MV medium (Lonza) supplemented with FCS to 10%. Human dermal fibroblasts (HDF) were cultivated in DMEM/Ham's F12 (1:1) 4 mm l-Glutamine 10% FCS.

Human Liver Sinusoidal Microvascular Endothelial Cells are from Cell Systems (ACBRI 566).

### PUVA treatment and stress-induced premature senescence

Subconfluent HDF were pretreated for 2 h with 40 ng mL^−1^ 8-methoxy-psoralen (8-MOP) in growth medium. For UVA, irradiation cells were incubated in PBS containing 40 ng mL^−1^ 8-MOP and irradiated with 9 J cm^−2^ (Hönle UV-technologie UVA-hand 25065 with a blacklight filter) controlled by a UV-meter (UV-Meter μC Nr.: 607439 FS UVA D1 E110 equipped with a sensor 16401). Nontreated and cells only irradiated with UVA served as a control.

The backs of C57BL/6 mice were shaved 1 day before PUVA treatment with an electric clipper. The mice were painted on their back with either 200 μL of 8-methoxypsoralen (8-MOP) in ethanol (1 mg mL^−1^) or 200 μL of vehicle (95% ethanol). UVA irradiation was performed after 30 min using a Sellamed 3000 UVA-1 therapy lamp (Sellas, Ennepetal, Germany) filtered for the emission at 340–400 nm at a distance of 20 cm from the dorsal skin of the mouse. The UVA dose used was 10 J cm^−2^. Mice were sacrificed 24 h and 48 h after a single PUVA exposure. Dorsal skin was excised and immediately frozen in liquid nitrogen for RNA analysis. All mice experiments were performed according to the rules of the Austrian law.

HUVECs (PD 20) were treated with 75 μm tert-butyl hydroperoxide (tBHP) in growth medium for one hour per day on 5 consecutive days as described by Dumont *et al*. (2000). After each treatment, the cells were washed twice with PBS, and normal growth media were added to the cells. RNA was isolated on day 10, where no proliferation was detected anymore.

### Senescence-associated beta-galactosidase

SA ß-gal staining was performed according to standard protocols described by Dimri *et al*. (1995).

### RNA isolation, quantification and quality control

Total RNA was isolated using Trizol reagent (Invitrogen, Life Technologies Ltd, Paisley, UK) according to the manufacturer's instructions. RNA concentration was determined by absorbance measurement on a Nanodrop ND-1000 spectrophotometer.

### RNA labelling, microarray hybridization and scanning

About 2 μg of total RNA was labelled according to the manufacturer's instructions with Hy3 or Hy5 fluorescent dyes (Exiqon miRCURY Hy3/Hy5 labelling kit). Corresponding Hy3 and Hy5 reactions were pooled and reduced to a total volume of 25 μL by vaccum centrifugation. Thereafter, reactions were mixed with 25 μL 2 × hybridization buffer and hybridization to Exiqon miRCURY LNA-arrays (v8.0) was performed for 16 h at 60 °C using a Tecan HS-400 station. Subsequently, microarrays were scanned with an Agilent Type B Scanner at 5 μm resolution, 16 bits per pixel and 100% PMT for both channels (532 and 635 nm). Feature intensities were extracted using GenePix 4.1 software, and the resulting GenePix result files (GPR-files) were processed under R 2.9.1 using the linear models for microarray data analysis package [LIMMA, (Smyth, 2004)]. Spot intensities were background corrected using the normexp function and normalized using local weighted linear regression (LOESS). For differential expression analysis, replicate spots of each miRNA probe were correlated, and linear models were fitted to the data (Smyth *et al*., 2005). Using a contrast matrix, paired comparisons of senescent vs. young HUVECs were calculated for each donor and tested for significance using moderated hypothesis tests (moderated t-statistic) as available in LIMMA. The resulting data were adjusted for multiple testing (p-adj.), by controlling the false discovery rate according to Benjamini and Hochberg (Benjamini *et al*., 2001). All miRNAs were ranked according to the adjusted *P*-values and a cut-off of p-adj. ≤ 0.05 was imposed.

### Northern blot analysis

About 10 μg of total RNA was used as input for Northern blot analysis adapted from (Lau, 2008). In brief, samples were run on a denaturing 15% polyacrylamide gel at 30 W for 1–2 h. Prior to transfer, equal loading was verified by staining with SYBR-Gold (Invitrogen, 1:10 000 in 0.5 × TBE) for 30 min and visualization on a Typhoon 9400 (Amersham, excitation 488 nm, emission 555 nm). Subsequently, RNA was transferred to positively charged nylon membranes (Pall Biodyne B 0.45 μm) on a semi-dry blotter and immobilized by UV-crosslinking with 100 mJ.

For hybridization, 20 pmol of anti-miR DNA-oligos (Invitrogen) was labelled for 1 h at 37 °C with 75–150 μCi γ-32P-ATP (6000 Ci mmol^−1^; GE-Healthcare, Uppsala, Sweden) using 1 μL T4 polynucleotide kinase (NEB, Ipswich, MA, USA). The probe was purified using G-25 columns (GE-Healthcare), mixed with hybridization buffer and hybridized to the membrane over night at 50 °C. For detection, membranes were exposed to PhosphoScreen (Kodak, Rochester, NY, USA) and scanned with a PhosphoImager (Molecular Dynamics Storm 860). Quantification was performed using ImageQuant 5.2 software after local median background subtraction.

**Table:** 

Probe name	Sequence
anti-hsa-miR-21	TCAACATCAGTCTGATAAGCTA
anti-hsa-miR-29a	AACCGATTTCAGATGGTGCTA
anti-hsa-miR-222	GAGACCCAGTAGCCAGATGTAGCT
anti-hsa-miR-31	CAGCTATGCCAGCATCTTGCC

### Quantitative realtime PCR

For quantification of miRNA expression, 10 ng of total RNA was reverse transcribed with the TaqMan MicroRNA Reverse Transcription Kit using specific primer according to the manufacturer's instructions (Applied Biosystems, Life Technologies Ltd, Paisley, UK). Quantitative Realtime PCR was performed on a Rotorgene realtime cycler (Corbett Research, Qiagen, Germantown, MD, USA) using TaqMan Universal PCR Master Mix, No AmpErase UNG as indicated by the manufacturer. Ct-values were determined at a threshold of 0.01 after dynamic tube normalization and slope correction. Relative quantification was performed by normalization to U6B snRNA using the 2^-ΔΔCt^ method (Livak & Schmittgen, 2001).

For quantification of mRNA expression, 100 ng of total RNA was reverse transcribed using Dynamo cDNA synthesis kit (Finnzymes, Thermo Scientific, Waltham, MA, USA). QRT-PCR was performed using SensiMix Plus SYBR-Green Mix (No UNG). Expression values were normalized to GAPDH and indicated as fold changes relative to control.

**Table:** 

Primer name	Sense primer	Antisense primer	Length
cdk2	CTCCTGGGCTGCAAATATTATTCCACAG	CCGGAAGAGCTGGTCAATCTCAGA	119 bp
p21^CIP1^	GGCGGCAGACCAGCATGACAGATT	GCAGGGGGCGGCCAGGGTAT	230 bp
p16	CAACGCACCGAATAGTTACG	AGCACCACCAGCGTGTC	177 bp
p53	GCTTTCCACGACGGTGAC	GCTCGACGCTAGGATCTGAC	97 bp
GAPDH	TGTGAGGAGGGGAGATTCAG	CGACCACTTTGTCAAGCTCA	210 bp

### Western blot

Proteinlysates were analysed under denaturing conditions on 4–12% NuPage Gels (Invitrogen) with 1 × MOPS as running buffer. Transfer was performed using the Novex XCell SureLock® Electrophoresis System (Invitrogen) according to manufacturer's instructions. Blocking was carried out with 3% nonfat dry milk in TPBS. PVDF membranes were probed with antibodies against CDK2 (sc-163, Santa Cruz Biotechnology), p21^CIP1^ (P1000-53, US Biologicals), CDC25A (sc-97, Santa Cruz Biotechnology), NFIB (ab11989; Abcam, Cambridge, UK), phosphoCDK2 (AJ1177b; Abgent, San Diego, CA, USA), beta-actin (A-5441, Sigma-Aldrich, St. Louis, MO, USA) and GAPDH (sc-25778, Santa Cruz, Dallas, TX, USA). Detection was performed on an Odyssey Infrared scanner.

### *In vitro* angiogenesis

The *in vitro* angiogenesis assay on matrigel (BD, Franklin Lakes, NJ, USA) was performed in 24-well cell culture plates according to the manufacturer's instruction using 5 × 10^4^ cells cm^−2^. Cells were incubated at 37 °C in a humidified atmosphere containing 5% CO_2_. Microscopy was performed 8-h postinoculation onto matrigel. Experiments were conducted in triplicates using random pipetting of the cells onto matrigel to avoid technical bias. Quantitative analysis of tubule complexes was performed by Angioquant software (Niemisto *et al*., 2005). Angioquant files were extracted with Excel to additionally perform paired Student's *t*-test to obtain *P*-values.

### Cell proliferation

MTT test was used to monitor metabolic activity as an indirect measure for proliferation. It was performed in 96-well culture plates using 8 × 10^3^ cells/0.3 cm^2^. 2 μg μL^−1^ MTT was added, and cells were incubated 4 h at 37 °C. Following, cells were lysed using 100 μL per well 10% SDS in 0.01 m HCL and incubated for 12 h at 37 °C. Measurement was taken by 570 nm/690 nm measurement on Tecan Infinite M200 reader (Tecan, Männedorf, Switzerland).

To monitor growth, pictures from randomly chosen microscopic fields were taken, and the cell number was determined. A minimum of 6 microscopic fields and at least 200 cells were counted.

### Apoptosis

For staining, the Annexin-V-fluos staining kit (Roche, Basel, Switzerland) was used. Cells were harvested and centrifuged at 170 g for 10 min and the pellet washed with Annexin-V binding buffer (10 mm Hepes/NaOH pH 7,4, 140 mm NaCl, 5 mm CaCl_2_). After centrifugation, the pellet was resuspended in Annexin-V/PI staining buffer (250 ng mL^−1^ propidium iodide, 200 ng mL^−1^ Annexin-V-Fluos, diluted in Annexin-V binding buffer). After 10 min incubation, samples were subjected to flow cytometric analysis. For compensation, cells were stained with either PI or Annexin-V alone. The analysis was performed using an excitation wavelength of 488-nm and a 600-nm emission filter for detection of PI (FL-3) and a 535-nm filter for Fluos (FL-1).

### Transfections

For investigations on miR-21, over-expression and CDC25A or NFIB knock-down HUVECs were used no later than PD 19. For miRNA transfection, 3 × 10^4^ cells cm^−2^ cells were seeded 24 h before transfection, and cells were transfected with 30 nm Pre-miR-21™miRNA Precursor Molecules (Ambion, Life Technologies Ltd, Paisley, UK). or Pre-miR™miRNA Precursor Molecules—Negative Control #2 (Ambion, Life Technologies Ltd, Paisley, UK) as a control using DharmaFECT 1 (Dharmacon, Thermo Scientific, Waltham, MA, USA) transfection reagent. For CDC25A or NFIB knock-down experiments, 1.5 × 10^4^ cells cm^−2^ were similarly transfected using 50 nm NFIB ON-TARGETplus SMARTpool siRNA (Dharmacon) or human CDC25A ON-TARGETplus siRNA (Dharmacon, Thermo Scientific, Waltham, MA, USA). As control ON-TARGETplus Nontargeting Pool siRNA (Dharmacon) was used. For miRNA knock-down experiments, anti-miR™miRNA Inhibitor targeting endogenous miR-21 and a nontargeting anti-miR™miRNA Inhibitor control were used at a concentration of 30 nm. Using siPort NeoFx (Ambion), 2 × 10^4^ cells were reverse transfected according to manufacturers instructions.

For stable over-expression of miR-21, we transduced HUVEC with lentiviral particles encoding hsa-mir-21 or a scrambled control miRNA. (GeneCopoeia # LP-HmiR0284-MR03-0200 and LP-CmiR0001-MR03-0200). Selection of stable clones was carried out using 1 μg mL^−1^ Puromycin. Transduction efficiency was measured as percentage of GFP expressing cells using a Gallios Flow Cytometer (Beckman Coulter, Brea, CA, USA).

### Dual-luciferase reporter gene assays

For Luciferase assays, HUVEC were used at no later PD than 10. For CDC25A, a 100 bp portion of the 3′UTR comprising the miR-21 target site (either wild-type or mutated) was cut out of a pLuc construct generously provided by Jian Yu (Ph.D., University of Pittsburg) and religated into psiCHECK2 (Promega, Madison, WI, USA). For NF1B, synthetic oligos comprising the miR-21-target site in the 3′UTR (wild-type and mutated) were cloned into psiCHECK2. HUVEC were cotransfected with 1 μg reporter plasmid and 15pmol miR precursor (control or miR-21) using the Nucleofector device (Amaxa) according to the manufacturer's instructions. Forty-eight hours post-transfection, cells were harvested and lysed by resuspending in Dual-Glo lysis buffer (Promega) and incubated for 5 min. Luciferase activity was measured on the Tecan Reader using the Dual-Glo Luciferase Assay System (Promega). Renilla luciferase signal was normalized against Firefly luciferase.

**Table:** 

Synthetic NF1B 3′UTR oligos	
NFIB_miR-21_TOPwt	TCGAGACTAGTgactttctagatgccTTAATATTTGCATGATAAGCTAgttttattggtttagGC
NFIB_miR-21_BOTTOMwt	GGCCGCctaaaccaataaaacTAGCTTATCATGCAAATATTAAggcatctagaaagtcACTAGTC
NFIB_miR-21_TOPmut	TCGAGACTAGTgactttctagatgccTTAATATTTGCATtgagttCTAgttttattggtttagGC
NFIB_miR-21_BOTTOMmut	GGCCGCctaaaccaataaaacTAGaactcaATGCAAATATTAAggcatctagaaagtcACTAGTC

### Statistics

If not indicated otherwise error bars represent mean ± standard deviation. When only two groups were compared, statistical analysis was performed using Student's *t*-test. anova and Bonferroni *post hoc* tests were performed where indicated. Statistical significance is indicated by asterisks representing *P*-values (*P < 0.05; **P < 0.01; ***P < 0.001). Data obtained from PUVA-treated mice were analysed using anova and Mann–Whitney *U*-test.

## Supporting Information

**Fig. S1.** Characterization of HUVEC senescence.

**Fig. S2.** Confirmation of miRNA micro-array profiling by QPCR and northern blot for a panel of additional, differentially expressed miRNAs in senescent HUVECs.

**Fig. S3.** Northern blot analysis confirms miR-21 up-regulation, which is not due to dicer up-regulation in senescent cells.

**Fig. S4.** Inhibitory effect of miR-21 on the proliferative potential of cultured cells does not involve p53 or p16 and is observed also in Hela cells.
