# Supplementary figures and images for: High levels of oncomiR-21 contribute to the senescence-induced growth arrest in normal human cells and its knock-down increases the replicative lifespan
Source: Aging Cell. 2013 Apr 19;12(3):446–58. doi: 10.1111/acel.12069 (PMC3864473; doi:10.1111/acel.12069)

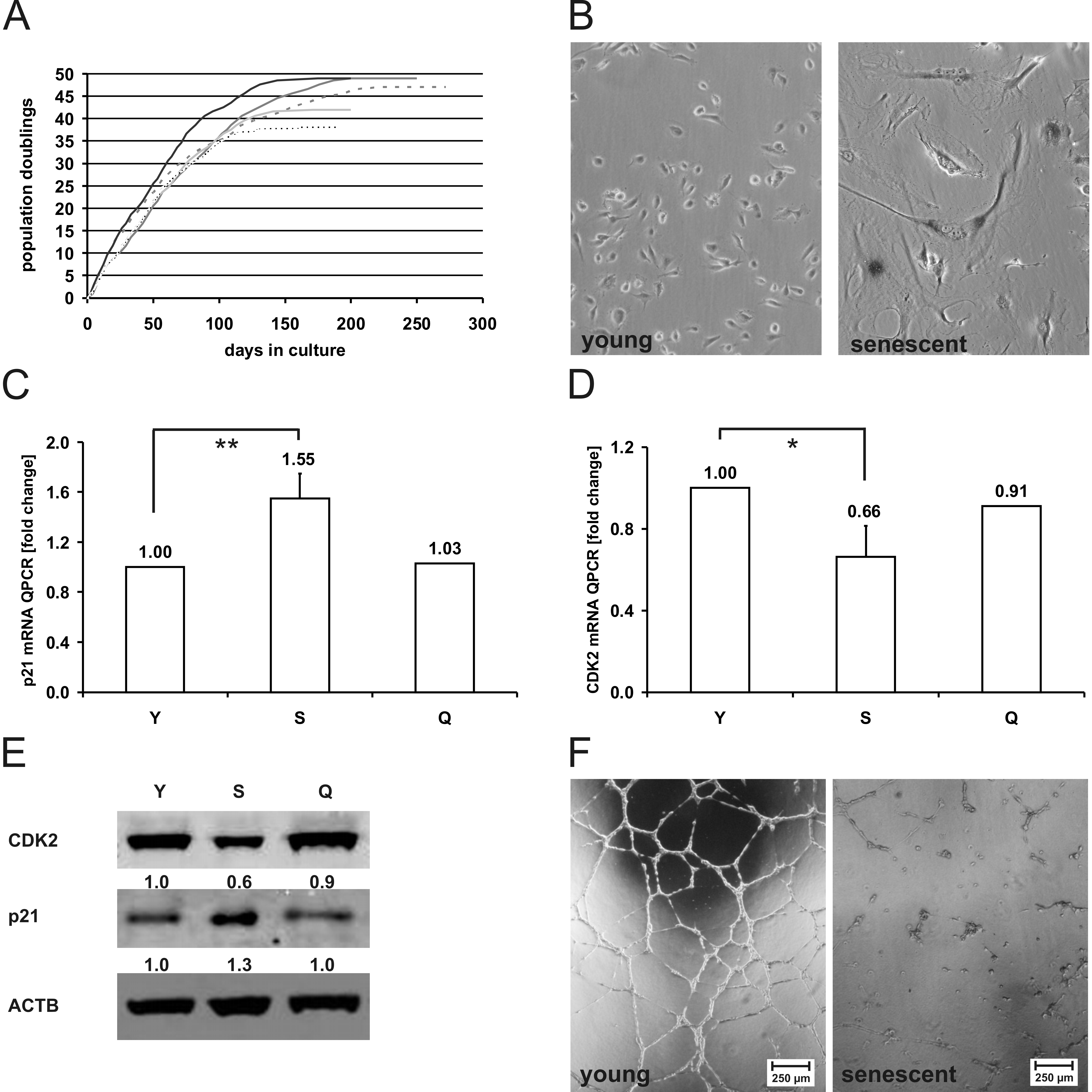

Supplement: Supplementary file 1 [file acel0012-0446-SD1.tif]

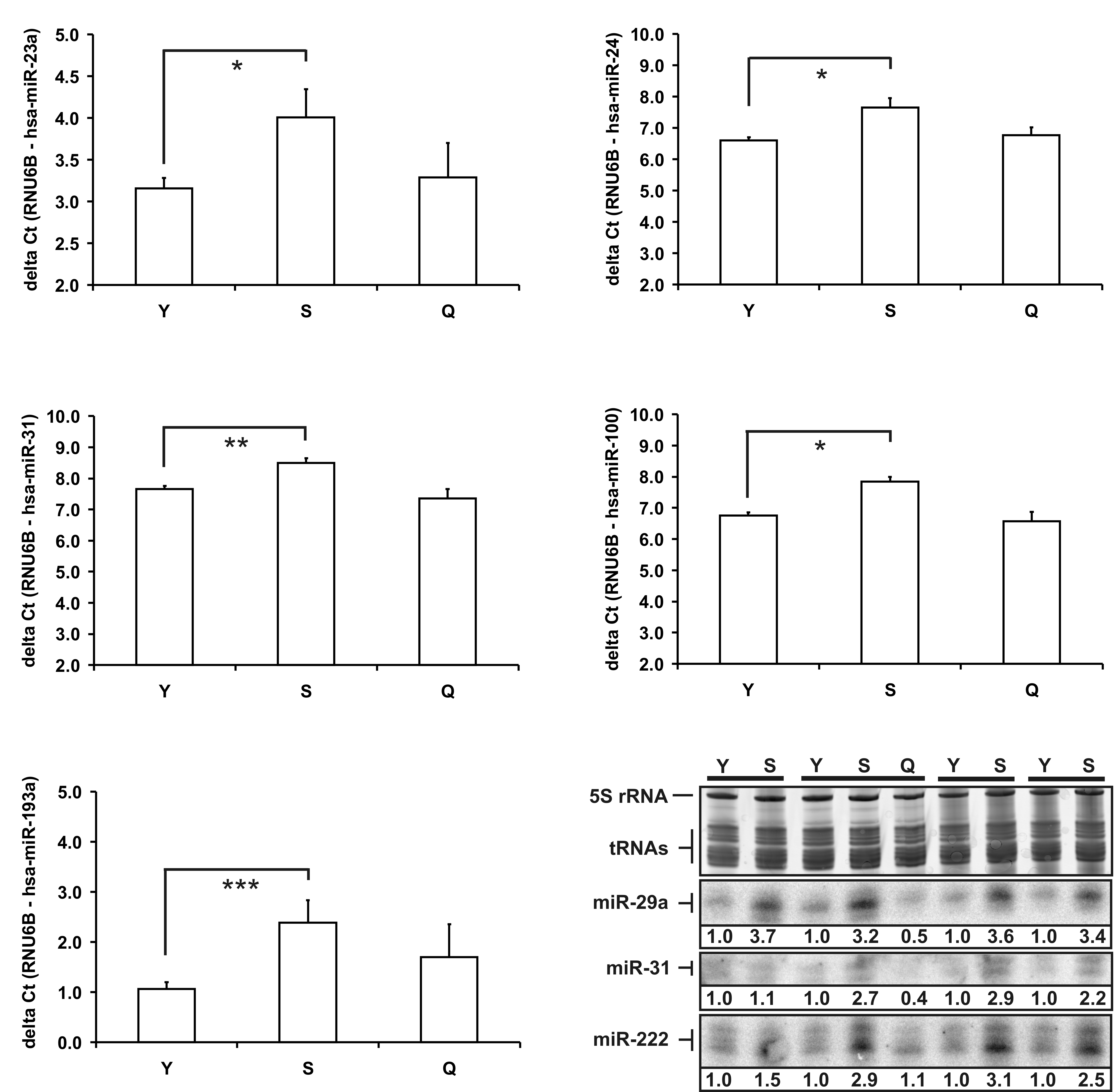

Supplement: Supplementary file 2 [file acel0012-0446-SD2.tif]

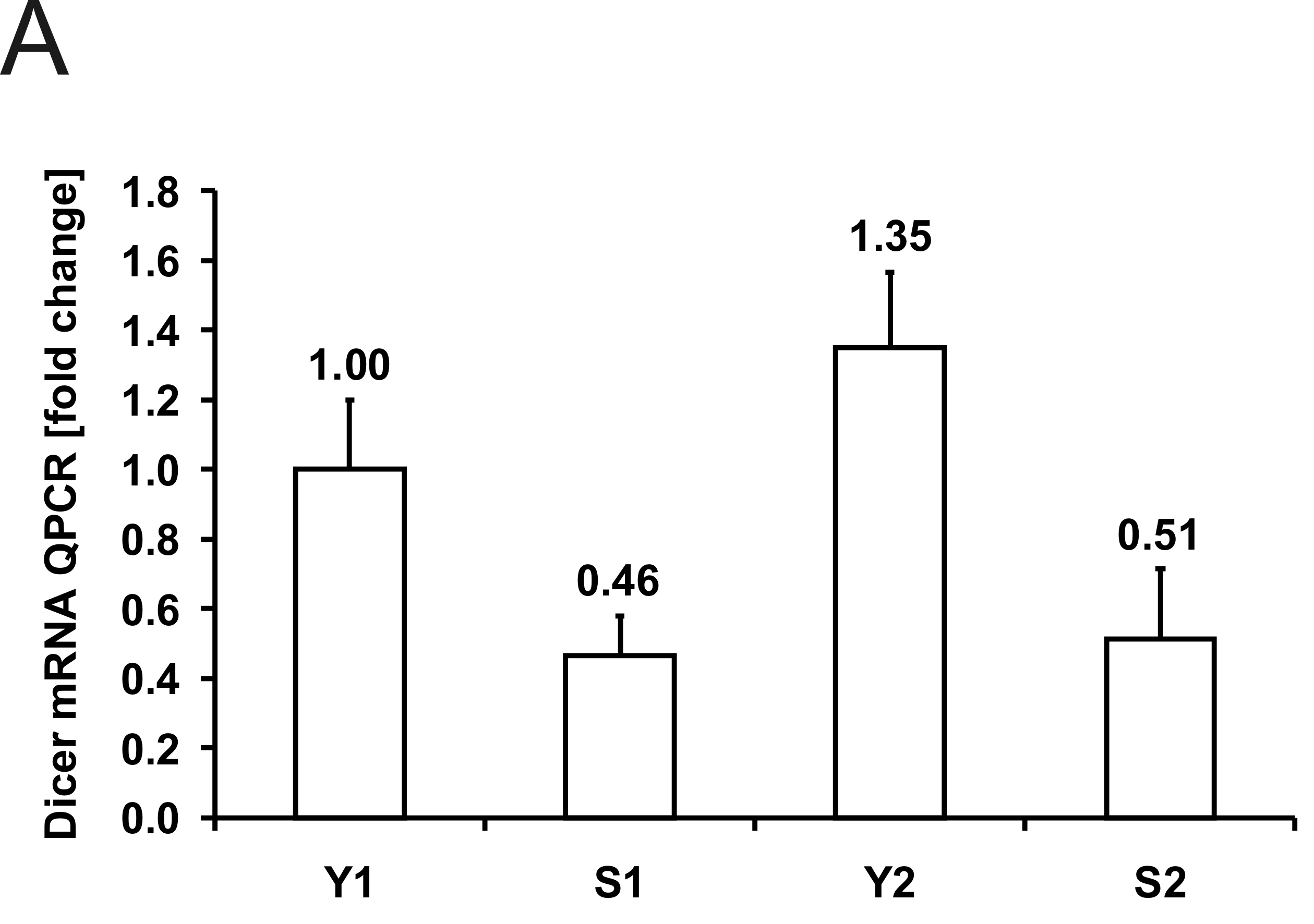

Supplement: Supplementary file 3 [file acel0012-0446-SD3.tif]

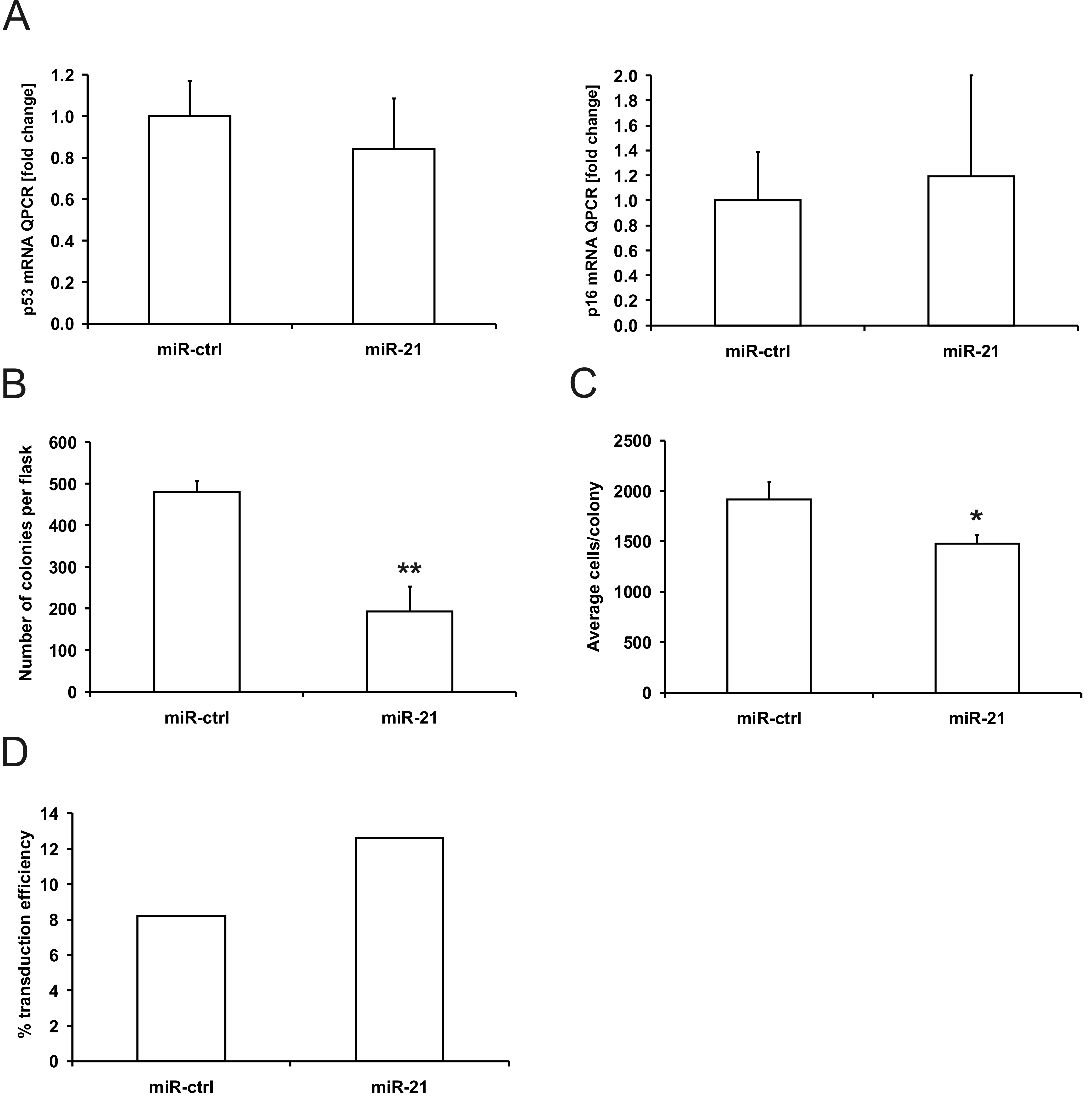

Supplement: Supplementary file 4 [file acel0012-0446-SD4.tif]
