# Supplementary material for: High levels of oncomiR-21 contribute to the senescence-induced growth arrest in normal human cells and its knock-down increases the replicative lifespan
Source: Aging Cell. 2013 Apr 19;12(3):446–58. doi: 10.1111/acel.12069 (PMC3864473; doi:10.1111/acel.12069)
Supplement: Supplementary file 5 [file acel0012-0446-SD5.doc]

# Supplemental figure legends

**Fig. S1 Characterization of HUVEC senescence.**

(A) Growth curves of 5 different HUVEC cell strains (3 male, 2 female). Cells reach senescence at population doubling 43 ± 6.

(B) SA-ß-gal staining, representative pictures of early passage (young) and senescent HUVECs.

(C) p21CIP1 (left) and CDK2 (right) mRNA expression was analysed by qPCR of young (Y), senescent (S) and quiescent (Q) HUVECs, fold changes normalized to GAPDH are indicated relative to young cells.

(D) p21CIP1 and CDK2 protein expression of young (Y), senescent (S) and quiescent (Q) HUVECs as analysed by Western blotting.

(E) Matrigel assay of young and senescent HUVECs showing reduced neoangiogenic potential of senescent HUVECs. Error bars represent mean ± standard deviation and statistical analysis was performed using ANOVA. P-values indicated represent *P < 0.05; ** P < 0.01.

**Fig. S2 Confirmation of miRNA micro-array profiling by QPCR and northern**

**blot for a panel of additional, differentially expressed miRNAs in senescent**

**HUVECs.**

For miR-23a, miR-24, miR100 and miR193a micro-array analysis was confirmed by taqman qPCR. For miR-29a and miR-222 micro-array analysis was confirmed by Northern blotting. For miR-31 micro-array analysis was confirmed by taqman qPCR as well as northern blot. qPCR data are displayed as delta Ct-values of young (Y), senescent (S), quiescent (Q) and

immortalized (I) HUVECs. For Northern blot, data fold changes to the corresponding young HUVEC strain are displayed.

**Fig. S3 Northern blot analysis confirms miR-21 up-regulation, which is not due to**

**dicer up-regulation in senescent cells.**

(A) MiRNA-processing RNAse Dicer mRNA expression of young (Y) and senescent (S) cells of two HUVEC strains analysed by qPCR and normalized to GAPDH. Relative fold changes to the corresponding senescent cells are displayed showing that young cells express

higher levels of Dicer mRNA.

**Fig. S4 Inhibitory effect of miR-21 on the proliferative potential of cultured cells does not depend on p53 or p16 and is observed also in Hela cells.**

(A) qPCR analysis of p53 (left panel) and p16 (right panel) mRNA levels of stable miR-21 over-expressing HUVEC at PD1 post-transduction. p53 and p16 expression levels were normalized to GAPDH and depicted relative to the average of four biological control replicates. Error bars represent standard deviations from 4 (control) or three (miR-21) biological replicates.

(B) Selection pressure was applied to Hela transduced with lentiviral particles over-expressing miR-21 or a nontargeting control shRNA. Colonies were counted on day 8 post-transduction. Error bars represent mean ± standard deviation obtained from three biological replicates. Statistical analysis was performed using Student’s t-test. P-values indicated represent *P < 0.05; **P < 0.01; ***P < 0.001.

(C) When selection was completed, cells were passaged into a fresh flask and cell numbers were determined. Average colony size was calculated by dividing cell number by number of colonies for each replicate separately. Error bars represent mean ± standard deviation obtained from three biological replicates. Statistical analysis was performed using Student’s t-test. P-values indicated represent *P < 0.05; **P < 0.01; ***P < 0.001.

(D) Hela were transduced with lentiviral particles overexpressing miR-21 or a non-targeting control shRNA. Transduction efficiency was determined by flow cytometric analysis of GFP-positive cells, which was expressed from the same promoter as the miR.
